# Technology-Assisted Cognitive Support for Older Adults With Mild Cognitive Impairment: Study Protocol for the DHEAL-COM COGNITIVE Quasi-Experimental Pilot Study

**DOI:** 10.2196/80233

**Published:** 2026-10-02

**Authors:** Roberta Bevilacqua, Elvira Maranesi, Elisa Felici, Rachele Alessandra Marziali, Arianna Margaritini, Arianna Sgolastra, Lucia Paciaroni, Alessandra Raccichini, Giuseppe Pelliccioni, Lucrezia Grassi, Carmine Tommaso Recchiuto, Fabrizia Lattanzio, Antonio Sgorbissa, Giulio Amabili

**Affiliations:** 1 IRCCS INRCA Istituto Nazionale di Ricovero e Cura per Anziani, Via Santa Margherita 5, Ancona, 60124, Italy, 0718004767 ext +39; 2 Dibris, Dipartimento di Informatica, Bioingegneria, Robotica e Ingegneria dei Sistemi, University of Genoa, Genova, Italy

**Keywords:** eHealth literacy, health literacy, older people, mild cognitive impairment, MCI, educational training, protocol, pilot study

## Abstract

**Background:**

Mild cognitive impairment (MCI) represents a transitional phase between normal aging and dementia. While no pharmacological treatments have proven effective, nonpharmacological interventions, such as cognitive stimulation and psychosocial support, have shown promise. Integrating digital tools and socially assistive robotics may improve engagement, personalization, and access to care, contributing to the preservation of cognitive function and overall well-being in older adults.

**Objective:**

The DHEAL-COM COGNITIVE pilot study aims to evaluate the feasibility, acceptability, and preliminary efficacy of a sociotechnological intervention that combines digital cognitive stimulation, social robot interaction, and group training in digital and health literacy to counteract cognitive decline.

**Methods:**

This is a single-blind, quasi-experimental feasibility trial involving 60 older adults with MCI, recruited from the Neurology and Alzheimer Units of IRCCS INRCA (INRCA – IRCCS Istituto Nazionale di Ricovero e Cura per Anziani). Participants were allocated into 2 groups: the experimental group received home-based cognitive training via the Brainer app, a month-long interaction with the NAO social robot, and weekly group sessions on eHealth literacy and cognitive stimulation; the control group received a well-being booklet with optional activities. Assessments were conducted at baseline, postintervention after 12 weeks, and follow-up after 3 months, using validated instruments, such as the Montreal Cognitive Assessment, the EQ-5D-5L, the Italian version of the eHealth Literacy Scale, the System Usability Scale, the unified theory of acceptance and use of technology, and the Psychological Well-Being Scale. Descriptive statistics will be used to summarize feasibility, usability, and acceptability outcomes, while preliminary differences over time will be explored using appropriate inferential analyses, acknowledging that the study is not powered to detect effectiveness.

**Results:**

Patient recruitment began in March 2025 and continued through September 2025. As of April 2026, 36 patients have been recruited, with 18 participants per group. The trial started in May 2025 and ran throughout the year. Results will focus on changes in cognitive performance, psychological well-being, quality of life, eHealth literacy, and user acceptability and usability of the technologies. Findings will be analyzed quantitatively and qualitatively and are expected to be published by the end of 2026.

**Conclusions:**

The DHEAL-COM COGNITIVE study proposes an innovative, integrated intervention for older adults with MCI, using digital platforms and social robotics to support cognitive health and autonomy. This pilot study represents a promising step toward a more integrated and technology-supported model of care for older adults with MCI.

## Introduction

### Background

As the global population continues to age, addressing the challenges associated with cognitive decline has become increasingly important [1]. Mild cognitive impairment (MCI) represents a critical stage in the continuum of cognitive decline, characterized by noticeable changes in cognitive abilities that are greater than expected for an individual’s age but do not yet interfere significantly with daily life activities [2]. If left unaddressed, MCI can progress to more severe conditions, such as Alzheimer disease and other forms of dementia, leading to a significant decline in quality of life and increased care needs [3].

The increasing prevalence of MCI and its potential progression to dementia necessitates early and effective intervention strategies [4]. These strategies often involve a combination of cognitive training exercises, lifestyle modifications, and medical management. Early intervention can delay the onset of more severe cognitive impairments, allowing individuals to maintain their independence and cognitive function for a longer period [5]. Furthermore, addressing MCI requires a holistic approach that considers not only cognitive aspects but also physical, emotional, and social components [6,7].

Currently, no drug has proven effective in treatment for MCI [8]. Because of the difficulty of pharmacological remedies in preventing or treating this condition, the most popular treatments for MCI are nonpharmacological [9,10]. Within this category, research has focused on cognitive training interventions, that is, a set of guided tasks that reflect specific cognitive functions (memory, attention, problem solving, executive functions) with the goal of improving or at least maintaining the specific function and with the ability to generalize results to daily life [11].

Traditional therapist-led, paper-pencil cognitive interventions have shown different results from interventions conducted with technology [12], the latter of which have several advantages. Indeed, technological interventions can be designed to be highly engaging and enjoyable, can provide immediate quantitative feedback and adjust task difficulty based on this feedback, and are actively accessible on portable digital devices [13]. For this reason, many traditional cognitive interventions have been adapted for use on current technological devices, such as smartphones, tablets, and computers, as they are considered a convenient alternative to traditional cognitive interventions [14]. In addition, supporting the cognitive well-being of older people with MCI involves creating a supportive environment that promotes their overall well-being. This includes providing emotional support, encouraging social interactions, and creating opportunities for meaningful engagement [15]. Such support can be provided through socially assistive robotics [16]. Designed to interact with humans on a social level, social robots provide companionship, cognitive stimulation, and support for daily activities by offering personalized interactions based on individual needs and preferences to help keep users engaged and motivated. Additionally, they can assist in monitoring health parameters, reminding users to take medications, and perform health-promoting activities [10]. The consistent and reliable support provided by social robots can reduce the burden on human caregivers, allowing them to focus on more complex tasks [17]. Finally, social robots can facilitate social connections by enabling virtual interactions with family members and friends, thereby mitigating feelings of loneliness and isolation, which are common among older adults with MCI [18].

Emerging evidence suggests that multimodal interventions that combine 2 or more approaches, such as cognitive training, physical training, or social activities, may have a greater impact on cognitive functioning in people with MCI compared to monomodal interventions [19]. However, despite this promising direction, the existing literature remains fragmented. Although studies have recently recognized the role of technology in MCI management, the focus has been on computerized cognitive training as a standalone intervention, without exploring the potential of technology to integrate cognitive, social, and functional dimensions of care [20]. Moreover, the impact of different combinations and dosages of nonpharmacological interventions for MCI remains insufficiently understood [10], limiting the development of structured, scalable, and sustainable intervention models.

Therefore, the present study aims to address these gaps by proposing and evaluating a combined, technology-based intervention that integrates cognitive training, social interaction, and digital literacy support, compared to a control condition lacking training elements. By explicitly targeting multiple dimensions of functioning and technology use, this protocol seeks to contribute to a more comprehensive understanding of how multimodal, technology-enhanced approaches can be designed for people with MCI.

### Goal of the Study

This paper presents the DHEAL-COM COGNITIVE field trial, a pilot study designed to assess the usability and acceptability of a cognitive stimulation system and the NAO robotic platform (Aldebaran) in patients with early-stage cognitive impairment and its preliminary evaluation of potential effects on cognitive functioning. Secondary and exploratory objectives include the preliminary evaluation of potential effects on well-being, quality of life, eHealth literacy, and social connectedness. DHEAL-COM COGNITIVE is an integrated ecosystem combining advanced digital infrastructures, cloud platforms, clinical data repositories, and open testing labs that facilitate the development, prototyping, and monitoring of innovative health solutions. In addition to home-based activities, participants will also take part in a group training program focused on developing digital and health-related skills, with the goal of promoting greater autonomy and familiarity with new technologies.

## Methods

### The Field Trial

The DHEAL-COM COGNITIVE trial is a feasibility pilot focused on usability and acceptability of a technological treatment for MCI with a quasi-experimental design. Outcome assessors responsible for data collection and evaluation were blinded to group allocation. Sixty older adults with MCI will be recruited for the study and allocated into 2 groups: the experimental group, which performed a stimulation using a specific software and a social robot; and the control group, which received a booklet containing information and activities on well-being. Patients in the control group were offered activities to perform at home in line with those proposed by the Neurology Unit of the IRCCS INRCA (INRCA – IRCCS Istituto Nazionale di Ricovero e Cura per Anziani), and they could complete these activities whenever they preferred. Figure 1 shows the design of the DHEAL-COM COGNITIVE study.

**Figure 1.**
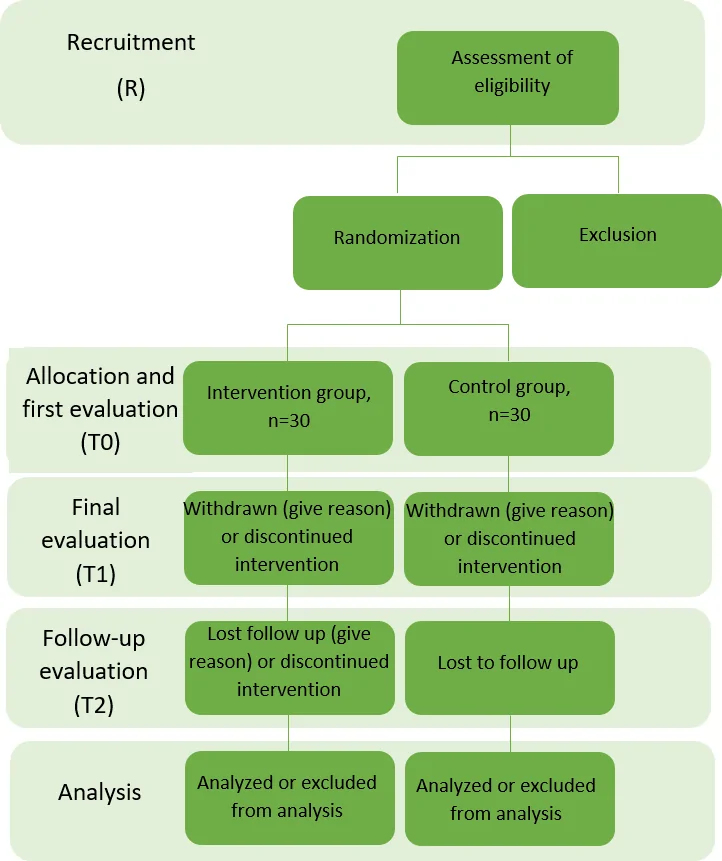
The study design of the DHEAL-COM COGNITIVE pilot.

### Pilot Study Setting

The experimentation involves 4 different phases: recruitment (R), first evaluation (T0) before the start of the trial, final evaluation (T1) at the end of the trial (after 12 weeks), and a follow-up evaluation (T2) 3 months after the end of the experiment.

### Participants

Participants were recruited from the Neurology Unit and Alzheimer Assessment Unit (Memory Clinic) and from the Alzheimer day care center of the IRCCS INRCA. After a reflection period of 2 weeks following reading the information letter and the consent form received by email, the participant communicated their wish to participate in the research. The doctor volunteering to carry out the inclusion visits first gave the participant the information letter and the 2 consent forms. After the participant and the doctor both proofread and signed the consent forms, the doctor attested the eligibility of the participant for research through an anamnesis, including the Montreal Cognitive Assessment (MoCA) [21-23] and the 5-item Geriatric Depression Scale [24]. This medical examination took place before the start of the experiment. Participants who signed the consent form, but whose inclusion criteria were not confirmed by the doctor, were not included in the research. The inclusion and exclusion criteria are reported in Textbox 1.

Textbox 1. **Inclusion and exclusion criteria for patients.**

The inclusion criteria were as follows:

- Aged 65 years and over
- Capacity to consent
- Already has a diagnosis of mild cognitive impairment
- Montreal Cognitive Assessment score 21‐27 [21]
- 5-item Geriatric Depression Scale score ≤1 [24]

The exclusion criteria were as follows:

- Failure to meet the inclusion criteria
- Use of active implant or nonimplant medical devices
- Allergy to nickel
- Concomitant participation in other studies
- Lack of written informed consent
- A myocardial infarction or stroke within 6 months
- Painful arthritis, spinal stenosis, amputation, painful foot lesions, or neuropathy limiting balance and mobility
- Uncontrolled hypertension
- Pacemaker or implantable cardioverter-defibrillator
- Metastatic cancer or immunosuppressive therapy
- Significant visual or hearing impairment that cannot be corrected

Following the visit with the doctor, the investigator contacted (by email or telephone) the participant to determine availability to organize the setup of home-based activities. When recruitment was complete, the investigator allocated participants into 2 groups: control group and experimental group. The allocation was done in an alternating manner (ABAB) according to the order of inclusion of the participants. Participants in the experimental group received the tablet with the Brainer app and the NAO robot. Participants in the control group received a booklet.

On the first day of the experiment (T0), a researcher visited the homes of all participants to carry out the initial assessment using the scales described below. In the experimental group, this was followed by an interview, and participants received training sessions in the study centers with the other experimental participants. Participants in the control group instead received a booklet containing information and activities related to well-being divided into 5 categories: cognitive activities, physical activities, psychological well-being, nutritional activities, and corrections to the paper-pencil exercises. Participants were free to use the booklet and carry out the proposed exercises at their discretion, as they might normally do in their daily lives.

On the last day of the experiment, ie, after 3 months (at ±15-day intervals depending on the participants’ availability), a final evaluation (T1) was administered to the participant. The researcher visited their home again, and they completed the questionnaires reported below. In addition, for the experimental group, a semistructured interview was conducted to collect the participant’s opinion. Finally, the researcher recovered all technological devices installed in the participant’s home (only applicable for the experimental group).

After 3 months past the end of the experiment, a follow-up evaluation (T2) was offered to the participant. The researcher visited their home again, and they completed various questionnaires, mentioned below.

### Equipment

In order to complete cognitive training, the participants of the experimental group received 2 technological devices.

First, the software Brainer (Brainer Srl), a web platform dedicated to cognitive rehabilitation exercises over 5 different domains, such as complex attention, executive function, learning and memory, language, and perceptual-motor skills. A dedicated tablet was provided to the participants to use the Brainer app.

Second, the social robot NAO V6 (Aldebaran), a fully programmable robot that can interact through its sensors and speech capabilities (ie, RGB camera, ultrasonic proximity sensors on the chest, 2 microphones, and tactile sensors on the head, hands, feet, and shoulders). NAO is also able to move thanks to multiple joints and actuators and perform multiple and very complex movements. It can also understand people’s language and emotions, as well as respond correctly to any request by speaking and making appropriate gestures.

In addition, as both NAO and Brainer require an internet connection, a web pocket equipped with a SIM card was given to the participant, to allow 4G connection of the devices. In this way, functioning of the equipment is guaranteed for the whole period of testing independently of the participant’s private home/mobile Wi-Fi.

The NAO robot was used with the CAIR (Cloud-based Autonomous Interaction with Robots) system, developed by the University of Genoa. The CAIR system is built on top of a client-server architecture; the client is executed on the robot, and it is connected to a server from which the robot gathers information on how to act (non)verbally with the user. The framework of the CAIR system is shown in Figure 2. In addition to the NAO robot, the mobile connection, the client software (CAIR), its interface for customization, and the computer server, the system integrates third-party software: Microsoft Speech Services, OpenAI GPT 3.5 Turbo, and GPT-4 Turbo (from now on called “OpenAI services”). All mentioned hardware is CE marked.

**Figure 2.**
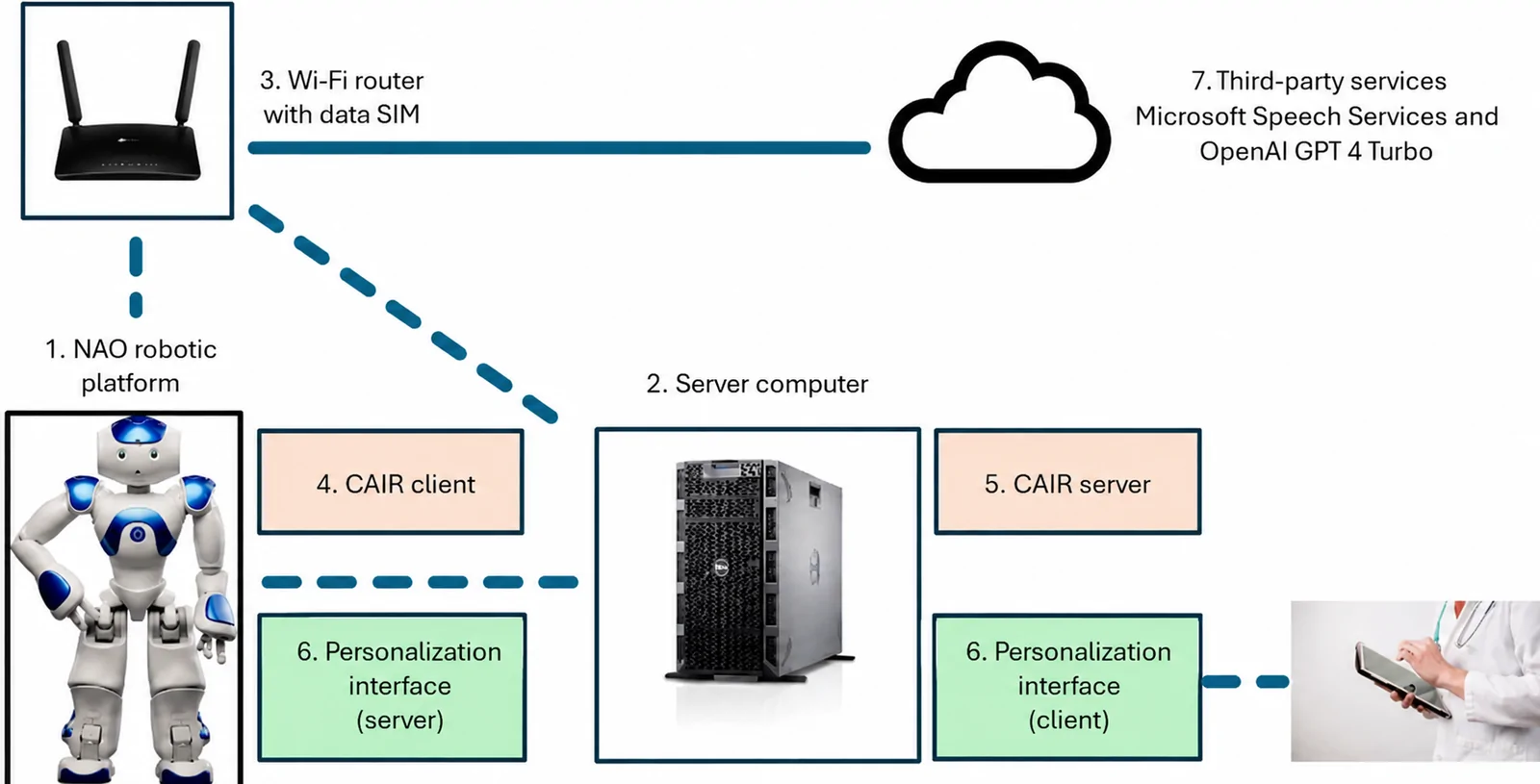
Framework of the CAIR (Cloud-based Autonomous Interaction with Robots) system.

The CAIR server software runs on the computer server and implements the functionality needed for the intervention under investigation. It receives information from the client CAIR about what the person said (information encrypted using https), accesses a system of internal rules aimed at implementing the intervention and OpenAI services to interpret the user’s sentences and produce an appropriate response in terms of phrases and actions, and returns instructions to the client CAIR about what the robot should say and do. Importantly, the CAIR server provides an alternative mechanism to the OpenAI services for creating response sentences, based on a rule-based system (Web Ontology Language) that contains sentences and sentence chunks composed in real time. This alternative mechanism, which allows full control over what the robot says because it uses only predefined, expert-validated sentences, is used whenever a critical topic of discussion is addressed (eg, the person’s health status, manifestation of physical or emotional discomfort, etc), allowing “safe” responses. These safe responses occur at the price of less variability in responses. Variability plays a key role in making the conversation interesting in the case of noncritical topics.

### Treatments

Participants were recruited from the Neurology Unit and Alzheimer Assessment Unit (Memory Clinic) and from the Alzheimer day care center of the IRCCS INRCA. The research team had the opportunity to communicate with participants of previous projects and initiatives, as well as with other people who might be interested in contributing to the project evaluations. This method made it possible to be in contact with clients they had met before and with whom a relationship of trust had been already established.

After the recruitment, the participant in the experimental group received a dedicated 1-to-1 training on the technological devices and procedure. In particular, they received the tablet with Brainer for starting cognitive training at home, and they followed the instruction of the neuropsychologist about the type of exercises to conduct in 5 different cognitive domains. Each participant was requested to use Brainer for 30 minutes a day, all along the duration of the experimentation. Moreover, the Brainer app allows 3 levels of increasing difficulty, adapting to the most appropriate difficulty for the individual participant. At each difficulty level, there are an average of 5 attempts (iterations). The exercises present different types of stimuli. Specifically, these are visual stimuli, such as pictures of animals, food and everyday objects, shapes, figures, and colors, and sounds, such as music and spoken words. The participant performing the exercises received feedback on his or her responses; the color green signaled the correct response, and the color red the incorrect response. The “home” version allowed homework to be assigned to the participant. This provided the opportunity to practice continuously (greater effectiveness), filling the time gap between sessions. All results generated from the participant’s home exercise were available in real time on the clinician’s account so that progress can be monitored.

In addition to daily cognitive stimulation, the participants were involved in group sessions once a week, in order to share their experiences and perform group activities, including memory exercises, such as recalling words, events, or images; logic games, such as puzzles or crosswords; or receive educational information. During these sessions, information was provided on disease and disease management, how to find health information online, and eHealth literacy. The group session included 15 participants at maximum and was held in the IRCCS INRCA YOUSE Lab facility. The group sessions were planned to last about 1 hour, once a week for 3 months.

In addition to computerized training and group sessions, each participant received the NAO robot for 1 month at their home. The participants were free to use the NAO robot to interact based on their main area of interests.

The robot-based experimental intervention conducted via a NAO robot that connects to a CAIR server was focused on personalized dialogue that took into account the individual’s interests. Specifically, through the humanoid robot’s verbal and nonverbal skills, it was possible to engage the individual in interactions and discussions on various topics of specific interest. Personalization of the robot’s behavior is done through a simple web interface for smartphones, tablets, or PCs, available to the experimenter. The personalization interface allows the user to enter data about the individual’s personal history, relationships, and preferences, and identify periods of the day when the robot will initiate the conversation (at other times of the day, the robot remains waiting for the person to initiate the conversation, available to his or her requests). Following the personalization procedure, the CAIR server then provides the robot with the information it needs to adapt to the individual’s interests, while also keeping track of the topics that resonate most with the individual interactively in order to calibrate future conversations accordingly. There are no limits in terms of time or duration of interactions; the user is free to interact whenever he or she desires.

Participants in the control group received a booklet containing information and activities on well-being. They were invited to do whatever they wished with the information booklet and the proposed exercises. The booklet contains information relating to psychology, cognition, and physical activity, in line with the general theoretical principles of healthy and active aging [25].

### Outcomes

The primary end point of the study constitutes both the usability and acceptability of an integrated intervention with technology and social robot and its preliminary impact on cognitive functioning of older people with MCI. Usability and acceptability will be assessed through the System Usability Scale (SUS) and the unified theory of acceptance and use of technology (UTAUT) and the preliminary impact on cognitive functioning through the MoCA, as described in Textbox 2.

Then, the field trials also focus on the improvement of psychological well-being, quality of life, eHealth literacy, acceptability, and usability, as shown in Textbox 3.

For these reasons, the protocol includes study-specific questions on demographics, quality of life, and psychological well-being, as well as physiological aspects and eHealth literacy level. All scales used are validated in the pilot sites’ languages and suitable for administration for the participants recruited in the study (interview guide in Multimedia Appendix 1). Table 1 summarizes the different tools used with participants in each phase of the study.

Textbox 2. **Primary outcomes of the study.**

- Usability and acceptability through System Usability Scale [26] and the unified theory of acceptance and use of technology [27]
- Preliminary impact on cognitive functioning through the Montreal Cognitive Assessment [21]

Textbox 3. **Secondary outcomes of the study.**

1. Improvement of quality of life of older people using the EQ-5D-5L [28]
2. Improvement of social connectedness of older people with the UCLA Loneliness Scale [29]
3. Improvement of mental well-being using the Psychological Well-Being Scale [30]
4. Improvement of eHealth literacy using the Italian version of the eHealth Literacy Scale [31]

**Table 1.** Tools and dimensions of the DHEAL-COM COGNITIVE protocol.

Dimension	Tool	R^a^	T0^b^	T1^c^	T2^d^
Overall health status	Clinical anamnesis	✔			
General information	Sociodemographics questionnaire	✔			
Cognitive status	MoCA^e^ [21]	✔		✔	✔
Psychological status	5-item Geriatric Depression Scale [24]	✔			
Frailty status	Clinical Frailty Scale [32]	✔			
Social connectedness	UCLA Loneliness Scale [29]		✔	✔	✔
Quality of life	EQ-5D-5L [28]		✔	✔	✔
Psychological well-being	Psychological Well-Being Scale [30]		✔	✔	✔
Acceptability	UTAUT^f^ [27]			✔	✔
Usability	SUS^g^ [26]			✔	✔
eHealth literacy	eHealth Literacy Scale (Italian version) [31]		✔	✔	✔

a R: recruitment.

b T0: first evaluation.

c T1: final evaluation.

d T2: follow-up evaluation.

e MoCA: Montreal Cognitive Assessment.

f UTAUT: unified theory of acceptance and use of technology.

g SUS: System Usability Scale.

### Statistical Analysis

To explore the preliminary efficacy of the intervention over time, several aspects will be taken into account. In the article by Park et al [33], the MoCA was assessed in 2 groups of patients with MCI, and the cases were treated by means of a virtual reality-based cognitive-motor rehabilitation (baseline and follow-up). Assuming an effect size of 50%, the total sample size required to capture this effect size was estimated to be 32 participants (16 per group), assuming a statistical power of 80%, a significance level of .05, 2 groups, and 2 repeated assessments (final assessment and a follow-up) in a within-between interactions ANOVA model. Even assuming a dropout rate of 20%, the total required number of participants would be 40 (20 in each arm).

The sample size calculation was based on the following statistical hypotheses:

- Null hypothesis (H₀): there is no significant difference in MoCA scores between the groups across the 2 time points (posttreatment and follow-up).
- Alternative hypothesis (H₁): there is a significant difference in MoCA scores between the groups across the 2 time points, particularly in favor of the group undergoing virtual reality-based cognitive–motor rehabilitation.

An effect size of 50% was chosen, consistent with the findings reported by Park et al [33], in which a clinically relevant improvement was observed in the intervention group compared to the control group. Given that our follow-up period is longer (3 months instead of 6 weeks as in the reference study), we decided to increase the total sample size to 60 participants (30 in each group) to ensure sufficient power to detect potential long-term changes in the primary outcome while accounting for a dropout rate of 20%.

The first step of the data analysis will deal with the description of the sample. Continuous variables will be reported as either mean and SD or median and IQR based on their distribution (assessed using Kolmogorov-Smirnov test). Categorical variables will be expressed as an absolute number and percentage. Changes in the primary and secondary outcomes across the 3 assessment time points will be analyzed using mixed-effects models including group, time, and group × time interaction terms. This approach allows for the analysis of repeated measures and accounts for within-subject correlations over time (eg, repeated measures ANOVA). When appropriate, additional exploratory analyses may be conducted using nonparametric tests in addition to simple descriptive statistics (means, medians, SDs, and IQRs as appropriate).

To verify the achievement of the primary end points (ie, SUS, UTAUT, and MoCA), subscale scores of the questionnaires will be calculated. Means and SDs or medians and IQRs of the scores will be reported according to their distribution. Correlation coefficients (Pearson for normally distributed variables, Spearman for nonnormally distributed variables) of the subscales with the other rating scales at each stage of the study, and the subscales with the main characteristics of the participants, will be calculated to check for potential determinants of higher acceptability. In addition, potential confounding variables collected through questionnaires and baseline assessments (eg, age, sex, education level, baseline cognitive performance, and other relevant clinical or psychosocial characteristics) will be explored as covariates. When appropriate, multivariable regression models will be used to adjust for these factors when analyzing changes in the primary outcome and secondary outcomes. This approach will allow for a more accurate estimation of the intervention effect while accounting for individual differences among participants. The analyses will be compared with the data from other partner countries. Descriptive statistical analyses will be performed on the quantitative data with SPSS (IBM SPSS Statistics) or RStudio (Posit PBC).

Regarding the qualitative data coming from the brief semistructured interviews, they will be analyzed through MAXQDA software (VERBI GmbH) for content analysis, in order to obtain frequency of reported themes and statements from the participants in line with the depth of the data collected.

### Dissemination Plan

The results of this study will be disseminated through peer-reviewed publications, conference presentations, and stakeholder engagement activities. Findings will also be shared with health care professionals and organizations involved in the care of older adults, with the aim of informing the development of technology-supported care models.

### Ethical Considerations

The study was approved by the Marche Territorial Ethics Committee (INRCA 6996 version 2 on February 26, 2025). The study protocol was recorded on ClinicalTrials.gov (NCT06984367).

The principles of the Declaration of Helsinki and Good Clinical Practice guidelines were adhered to. All participants in this study provided written informed consent. Personal data collected during the trial will be handled and stored in accordance with the General Data Protection Regulation 2018. For this purpose, data will be anonymized at the end of the study. Use of the study data will be controlled by the principal investigator. All data and documentation related to the trial will be stored in accordance with applicable regulatory requirements, and access to data will be restricted to authorized study personnel. Trial conduct will be monitored through an annual institutional review focusing on recruitment rates and study progress. Given the low-risk nature of the study, no independent data monitoring committee or interim analyses are planned.

## Results

Participant recruitment started in March 2025 and continued throughout the year. As of April 2026, 36 participants have been recruited, with 18 participants per group. At the end of recruitment, 30 of the total 60 older adults with MCI will be assigned to the experimental group and 30 to the control group. The trial started in May 2025 and continued throughout the rest of the year. The feasibility of the DHEAL-COM COGNITIVE ecosystem will be demonstrated based on participants’ opinions of the usability and acceptability of the technological intervention, as well as its preliminary efficacy in improving or maintaining cognitive functioning. Additionally, the effects on participants’ quality of life, social connectedness, psychological well-being, and eHealth literacy will contribute to the eventual determination of the intervention’s efficacy. The preliminary efficacy of the DHEAL-COM COGNITIVE study will be demonstrated in a separate publication expected to be published by the end of 2026.

## Discussion

### Principal Findings

The DHEAL-COM pilot study addresses a public health challenge, namely the prevention and management of cognitive decline among older adults with MCI. By integrating cognitive stimulation technologies with social robotics and digital health literacy training, the study primarily aims to evaluate usability and acceptability, while exploring potential trends in cognitive, psychological, eHealth literacy, and quality-of-life outcomes.

In fact, previous studies suggest that computerized cognitive training may be beneficial for people with MCI [11] and that multidomain interventions may be more effective than single-component approaches as they target multiple aspects [10,19]. This protocol contributes to this field by proposing a multimodal intervention that combines cognitive training, social robotics, and eHealth literacy, while assessing usability and acceptability for real-world implementation.

### Strengths and Limitations

This study has several strengths. First, it adopts a multidomain, person-centered design, combining home-based cognitive training (via the Brainer app), social engagement and companionship (via the NAO robot), and group-based educational sessions focused on health and digital literacy. This integrated model not only targets cognitive domains but also addresses social connectedness, emotional well-being, and autonomy, thereby offering a response to the need to develop multimodal interventions that specify components, methods, and timelines implemented [21]. Second, the inclusion of a control group with minimal intervention (ie, a well-being booklet) ensures a clear comparison, allowing researchers to isolate the specific effects of the technological and robotic intervention. Moreover, through qualitative interviews and scales such as UTAUT and SUS, the study aims to understand not only whether the intervention is effective, but also whether it is usable and acceptable for older adults. This is essential for informing potential scalability and integration into routine clinical and community-based care settings. Finally, the home-based design enhances ecological validity and reflects real-world conditions, supporting the potential scalability and sustainability of the intervention.

However, some limitations should be acknowledged. As a pilot feasibility study, the sample size is limited and not powered to detect statistically significant effects on clinical outcomes. In addition, the use of the MoCA may be subject to test-retest effects and have limited sensitivity in detecting short-term changes. Another potential limitation is the complexity of the intervention, which makes it difficult to disentangle the individual contribution of each component (cognitive training, robot interaction, group sessions). Future studies may consider factorial designs or component analyses to better understand mechanisms of action. Finally, another potential limitation concerns the imbalance in intervention intensity between experimental and control groups. This may introduce attention and expectancy effects. Future studies could include active control conditions.

### Future Directions

The findings of this pilot study could inform the design of future larger-scale randomized controlled trials aimed at evaluating the efficacy of the intervention. Future research could explore the mechanisms linking the components of the intervention to the outcomes (eg, the effect of using the NAO on perceived loneliness, measured using the UCLA Loneliness Scale) and the long-term sustainability of the observed effects. Additionally, further work is needed to better understand factors influencing technology adoption among older adults.

### Conclusions

In conclusion, this pilot protocol represents a promising step toward a more integrated and technology-supported model of care for older adults with MCI. The knowledge gained from this study will help provide evidence for the design of larger studies and the implementation of digital health and robotics solutions in aging care settings.

## Supplementary material

10.2196/80233 Multimedia Appendix 1 Interview guide.

## Abbreviations

CAIR: Cloud-based Autonomous Interaction with Robots
IRCCS INRCA: INRCA – IRCCS Istituto Nazionale di Ricovero e Cura per Anziani
MCI: mild cognitive impairment
MoCA: Montreal Cognitive Assessment
R: recruitment
SUS: System Usability Scale
UTAUT: unified theory of acceptance and use of technology
