# Supplementary material for: Technology-Assisted Cognitive Support for Older Adults With Mild Cognitive Impairment: Study Protocol for the DHEAL-COM COGNITIVE Quasi-Experimental Pilot Study
Source: JMIR Res Protoc. 2026 Oct 2;15:e80233. doi: 10.2196/80233 (PMC13632969; doi:10.2196/80233)
Supplement: Supplementary file 1 [file resprot-v15-e80233-s001.pdf]

DHEAL COM COGNITIVE

CASE REPORT FORM

Identification code:
Date of compilation:
Name of the interviewer:

Recruitment (R)

Socio-demographic characteristics

1. Date of birth: ____________

2. Gender:

- ☐ Male
- ☐ Female
- ☐ Other
- ☐ Prefer to not specify

3. Please specify what is your marital status at present:

- ☐ Married (living with the spouse/wife)
- ☐ In a relationship
- ☐ Separated (married, but living separately)
- ☐ Divorced
- ☐ Single
- ☐ Widowed

4. Can you indicate which of the following education level have you reached?

- ☐ No education
- ☐ Primary education
- ☐ Secondary education
- ☐ Tertiary education (university or further education level)

5. Please indicate your present working situation:

- ☐ Retired
- ☐ Working full time
- ☐ Working part-time
- ☐ Unemployed

Montreal Cognitive Assessment (MoCA)

MoCA COGNITIVE ASSESSMENT

Version 8.1 English

Name:
Education:
Sex:
Date of birth:
DATE:

Section	Task	Points
**VISUOSPATIAL / EXECUTIVE**	Trail making [ ]; Copy cube [ ]; Draw CLOCK (Ten past eleven) (3 points) [ ] Contour [ ] Numbers [ ] Hands	__/5

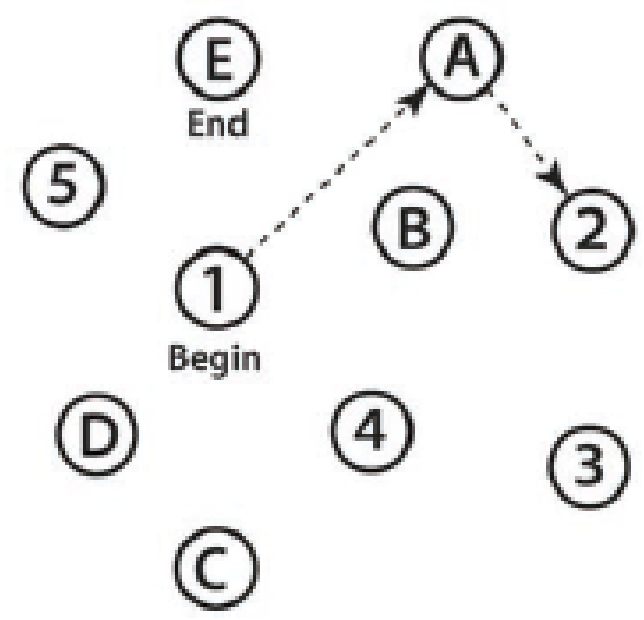

[ ]

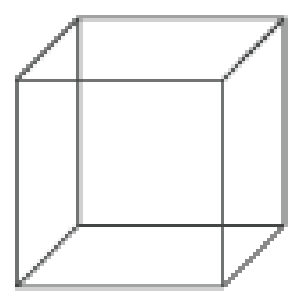

Copy cube

[ ]

Draw CLOCK (Ten past eleven)
(3 points)

[ ] Contour [ ] Numbers [ ] Hands

__/5

NAMING

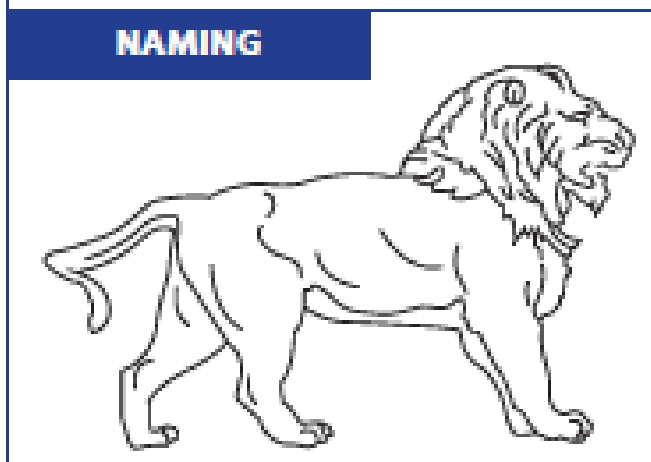

[ ]

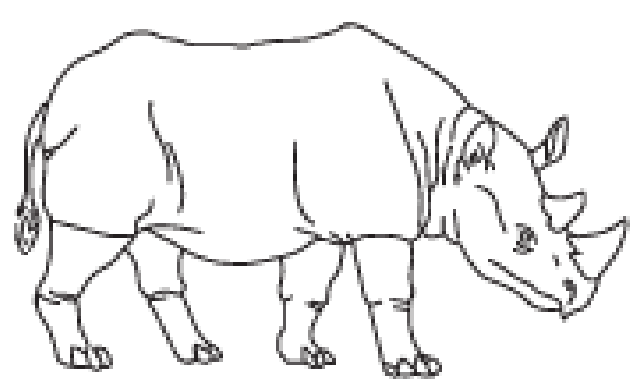

[ ]

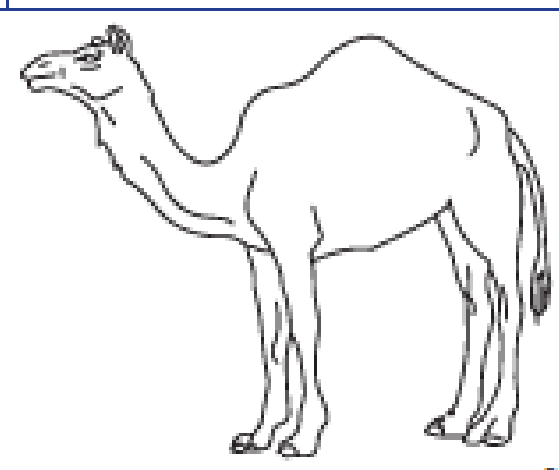

[ ]

__/3

MEMORY Read list of words, subject must repeat them. Do 2 trials, even if 1st trial is successful. Do a recall after 5 minutes.

	FACE	VELVET	CHURCH	DAISY	RED
1ST TRIAL					
2ND TRIAL					

NO POINTS

ATTENTION Read list of digits (1 digit/ sec.).
Subject has to repeat them in the forward order. [ ] 2 1 8 5 4
Subject has to repeat them in the backward order. [ ] 7 4 2
__/2

Read list of letters. The subject must tap with his hand at each letter A. No points if ≥ 2 errors
[ ] F B A C M N A A J K L B A F A K D E A A A J A M O F A A B
__/1

Serial 7 subtraction starting at 100. [ ] 93 [ ] 86 [ ] 79 [ ] 72 [ ] 65
4 or 5 correct subtractions: **3 pts**, 2 or 3 correct: **2 pts**, 1 correct: **1 pt**, 0 correct: **0**
__/3

LANGUAGE Repeat: I only know that John is the one to help today. [ ]
The cat always hid under the couch when dogs were in the room. [ ]
__/2

Fluency: Name maximum number of words in one minute that begin with the letter F. [ ] _____ (N ≥ 11 words)
__/1

ABSTRACTION Similarity between e.g. orange - banana = fruit [ ] train - bicycle [ ] watch - ruler
__/2

DELAYED RECALL

(MIS)		FACE	VELVET	CHURCH	DAISY	RED	
	Has to recall words WITH NO CUE	[ ]	[ ]	[ ]	[ ]	[ ]	Points for UNCUED recall only
X3							
X2	Category cue						MIS = ____/15
X1	Multiple choice cue						

Memory Index Score (MIS)

__/5

ORIENTATION [ ] Date [ ] Month [ ] Year [ ] Day [ ] Place [ ] City
__/6

© MoCA Test Inc.
Created by Z. Nasreddine MD

www.mocacognition.com

Training and Certification are required to ensure accuracy.

MIS: /15
(Normal ≥ 26/30)
Add 1 point if ≤ 12 yr edu

TOTAL __/30

Five-items Geriatric Depression Scale

Choose the best answer for how you have felt over the past week:

Are you basically satisfied with your life?.	Yes	No
Do you often get bored?	Yes	No
Do you often feel helpless?	Yes	No
Do you prefer to stay at home rather than going out and doing new things?	Yes	No
Do you feel pretty worthless the way you are now?	Yes	No

Total: __/5

Clinical Frailty Scale (CFS)

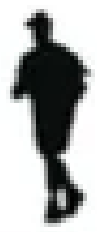	1. Very Fit — People who are robust, active, energetic and motivated. These people commonly exercise regularly. They are among the fittest for their age.
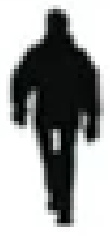	2. Well — People who have **no active disease symptoms** but are less fit than category 1. Often, they exercise or are **very active occasionally**, e.g. seasonally.
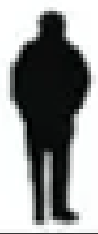	3. Managing Well — People whose **medical problems are well controlled**, but are **not regularly active** beyond routine walking.
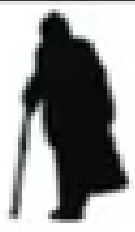	4. Vulnerable — While **not dependent** on others for daily help, often **symptoms limit activities**. A Common complaint is being "slowed up', and/or being tired during the day.
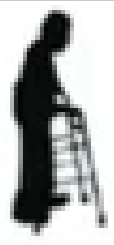	5. Mildly Frail — These people often have **more evident slowing**, and need help in **high order IADLs** (finances, transportation, heavy housework, medications). Typically, mild frailty progressively impairs shopping and walking outside alone, meal preparation and housework.
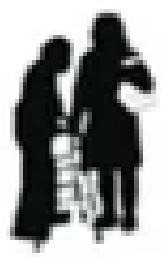	6. Moderately Frail — People need help with **all outside activities** and with **keeping house**. Inside, they often have problems with stairs and **need help with bathing** and might need minimal assistance (cuing, standby) with dressing.
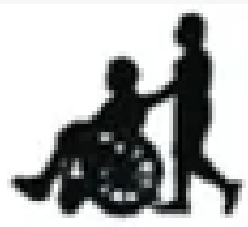	7. Severely Frail — **Completely dependent for personal care**, from whatever cause (physical or cognitive). Even so, they seem stable and not at high risk of dying (within ~ 6 months).
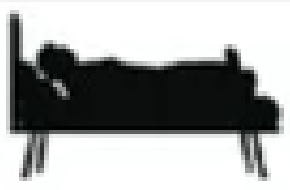	8. Very Severely Frail — Completely dependent, approaching the end of life. Typically, they could not recover even from a minor illness.
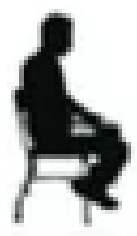	9. Terminally Ill - Approaching the end of life. This category applies to people with **a life expectancy <6 months**, who are **not otherwise evidently frail**.

Baseline Assessment (T0)

Identification code:
Date of compilation:
Name of the interviewer:

UCLA Version 3 Loneliness Scale

UCLA Version 3 Loneliness Scale

Instructions: The following statements describe how people sometimes feel. For each statement, please indicate how often you feel the way described by placing a check in the space provided. Here is an example: How often do you feel happy? If you never felt happy, you would check "never"; if you always feel happy, you would check "always."

	NEVER 1	RARELY 2	SOMETIMES 3	ALWAYS 4
*1. How often do you feel that you are "in tune" with the people around you?				
2. How often do you feel that you lack companionship?				
3. How often do you feel that there is no one you can turn to?				
4. How often do you feel alone?				
*5. How often do you feel part of a group of friends?				
*6. How often do you feel that you have a lot in common with the people around you?				
7. How often do you feel that you are no longer close to anyone?				
8. How often do you feel that your interests and ideas are not shared by those around you?				
*9. How often do you feel outgoing and friendly?				
*10. How often do you feel close to people?				
11. How often do you feel left out?				
12. How often do you feel that your relationships with others are not meaningful?				
13. How often do you feel that no one really knows you well?				
14. How often do you feel isolated from others?				
*15. How often do you feel you can find companionship when you want it?				
*16. How often do you feel that there are people who really understand you?				
17. How often do you feel shy?				
18. How often do you feel that people are around you but not with you?				
*19. How often do you feel that there are people you can talk to?				
*20. How often do you feel that there are people you can turn to?				

Scoring: Items that are asterisked should be reversed (i.e., 1 4, 2 3, 3 2, 4 1), and the scores for each item then summed together. Higher scores indicate greater degrees of loneliness. From Russell DW: UCLA Loneliness Scale (Version 3): reliability, validity, and factor structure, *J Pers Assess* 66:20-40, 1996.

IT-eHEALS

eHealth Literacy Scale

I would like to ask you for your opinion and about your experience using the Internet for health information. For each statement, tell me which response best reflects your opinion and experience *right now*.

1. How **useful** do you feel the Internet is in helping you in making decisions about your health?

o1	o2	o3	o4	o5
Not useful at all	Not useful	Unsure	Useful	Very Useful

2. How **important** is it for you to be able to access health resources on the Internet?

o1	o2	o3	o4	o5
Not important at all	Not important	Unsure	Important	Very important

3. I know **what** health resources are available on the Internet

1) o Strongly Disagree
2) o Disagree
3) o Undecided
4) o Agree
5) o Strongly Agree

4. I know **where** to find helpful health resources on the Internet

1) o Strongly Disagree
2) o Disagree
3) o Undecided
4) o Agree
5) o Strongly Agree

5. I know **how** to find helpful health resources on the Internet

1) o Strongly Disagree
2) o Disagree
3) o Undecided
4) o Agree
5) o Strongly Agree

6. I know **how to use** the Internet to answer my questions about health

1) o Strongly Disagree
2) o Disagree
3) o Undecided
4) o Agree
5) o Strongly Agree

7. I know how to use **the health information** I find on the Internet to help me

1) o Strongly Disagree
2) o Disagree
3) o Undecided
4) o Agree
5) o Strongly Agree

8. I have the skills I need to **evaluate** the health resources I find on the Internet

1) o Strongly Disagree
2) o Disagree
3) o Undecided
4) o Agree
5) o Strongly Agree

9. I can tell **high quality** health resources from **low quality** health resources on the Internet

1) o Strongly Disagree
2) o Disagree
3) o Undecided
4) o Agree
5) o Strongly Agree

10. I feel **confident** in using information from the Internet to make health decisions

1) o Strongly Disagree
2) o Disagree
3) o Undecided
4) o Agree
5) o Strongly Agree

Thank you!

** Note: Questions #1 and #2 are recommended as supplementary items for use with the eHEALS to understand consumer's interest in using eHealth in general. These items are not a formal part of theeHealth Literacy scale, which comprises questions #3-10.*

EQ-5D-5L

Under each heading, please tick the ONE box that best describes your health TODAY

MOBILITY

I have no problems in walking about ☐

I have slight problems in walking about ☐

I have moderate problems in walking about ☐

I have severe problems in walking about ☐

I am unable to walk about ☐

SELF-CARE

I have no problems washing or dressing myself ☐

I have slight problems washing or dressing myself ☐

I have moderate problems washing or dressing myself ☐

I have severe problems washing or dressing myself ☐

I am unable to wash or dress myself ☐

USUAL ACTIVITIES *(e.g. work, study, housework, family or leisure activities)*

I have no problems doing my usual activities ☐

I have slight problems doing my usual activities ☐

I have moderate problems doing my usual activities ☐

I have severe problems doing my usual activities ☐

I am unable to do my usual activities ☐

PAIN / DISCOMFORT

I have no pain or discomfort ☐

I have slight pain or discomfort ☐

I have moderate pain or discomfort ☐

I have severe pain or discomfort ☐

I have extreme pain or discomfort ☐

ANXIETY / DEPRESSION

I am not anxious or depressed ☐

I am slightly anxious or depressed ☐

I am moderately anxious or depressed ☐

I am severely anxious or depressed ☐

I am extremely anxious or depressed ☐

VAS

To help people say how good or bad a health state is, we have drawn a scale (rather like a thermometer) on which the best state you can imagine is marked 100 and the worst state you can imagine is marked 0.

We would like you to indicate on this scale how good or bad your own health is today, in your opinion. Please do this by drawing a line from the box below to whichever point on the scale indicates how good or bad your health state is today.

Your own health state today

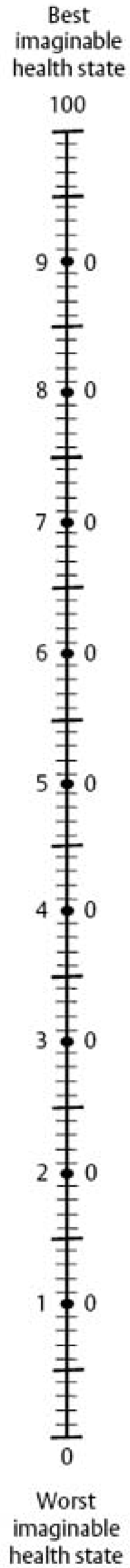

PWB - 18 items

Please indicate your degree of agreement to the following sentences.

1 = Strongly disagree 6 = Strongly agree

Maintaining close relationships has been difficult and frustrating for me.	1	2	3	4	5	6
The demands of everyday life often get me down.	1	2	3	4	5	6
In many ways, I feel disappointed about my achievements in life.	1	2	3	4	5	6
I like most aspects of my personality.	1	2	3	4	5	6
I have not experienced many warm and trusting relationships with others.	1	2	3	4	5	6
I judge myself by what I think is important, not by the values of what others think is important.	1	2	3	4	5	6
In general, I feel I am in charge of the situation in which I live.	1	2	3	4	5	6
I am quite good at managing the many responsibilities of my daily life.	1	2	3	4	5	6
I tend to be influenced by people with strong opinions.	1	2	3	4	5	6
I gave up trying to make big improvements or changes in my life a long time ago.	1	2	3	4	5	6
I think it is important to have new experiences that challenge how you think about yourself and the world.	1	2	3	4	5	6
I live life one day at a time and don't really think about the future.	1	2	3	4	5	6
I am not afraid to voice my opinions, even when they are in opposition to the opinions of most people.	1	2	3	4	5	6
Some people wander aimlessly through life, but I am not one of them.	1	2	3	4	5	6
Some people wander aimlessly through life, but I am not one of them.	1	2	3	4	5	6
For me, life has been a continuous process of learning, changing and growth.	1	2	3	4	5	6
People would describe me as a giving person, willing to share my time with others.	1	2	3	4	5	6
I sometimes feel as if I've done all there is to do in life.	1	2	3	4	5	6
When I look at the story of my life, I am pleased with how things have turned out.	1	2	3	4	5	6

Final Treatment Assessment (T1)

Identification code:
Date of compilation:
Name of the interviewer:

Montreal Cognitive Assessment (MoCA)

MoCA COGNITIVE ASSESSMENT

Version 8.1 English

Name:
Education:
Sex:
Date of birth:
DATE:

		POINTS
**VISUOSPATIAL / EXECUTIVE**		

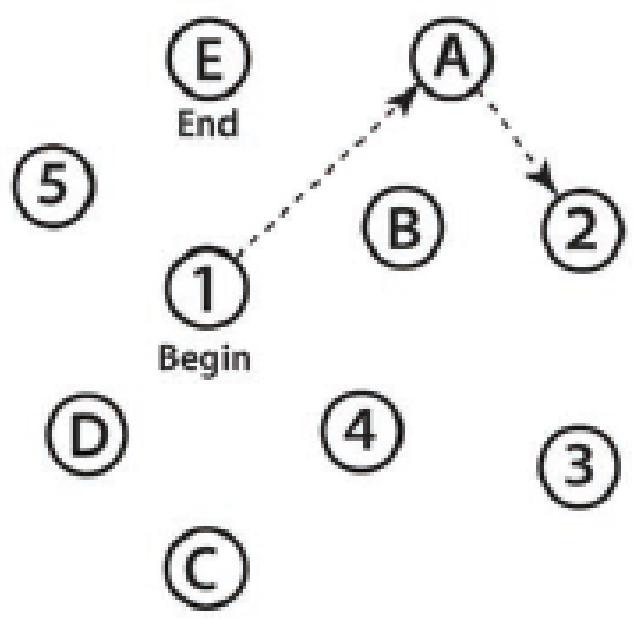

[ ]

Copy cube

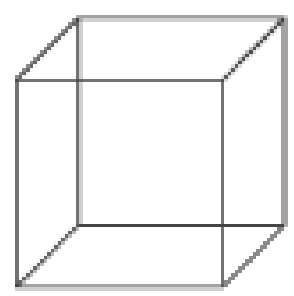

[ ]

Draw CLOCK (Ten past eleven)
(3 points)

[ ] Contour [ ] Numbers [ ] Hands

__/5

NAMING

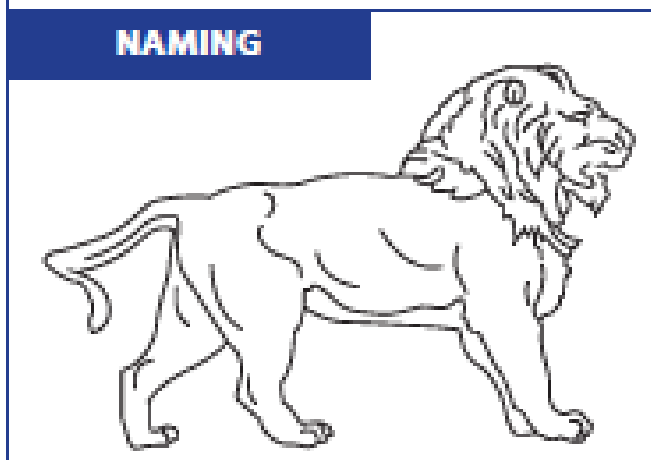
[ ]

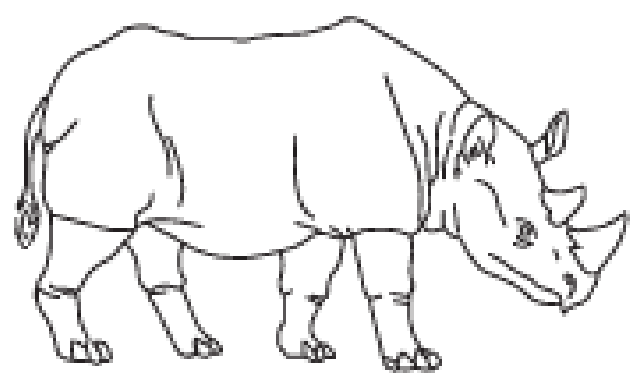
[ ]

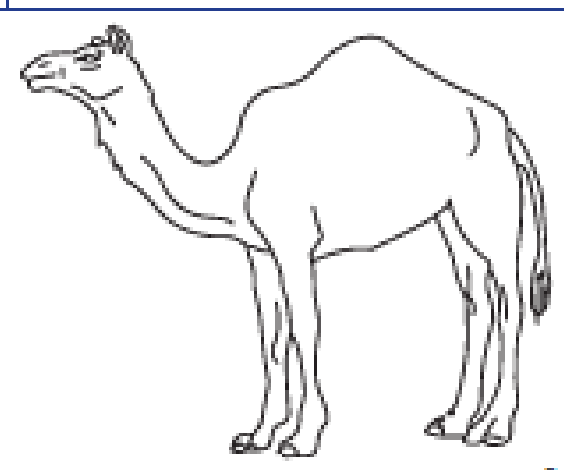
[ ]

__/3

MEMORY

Read list of words, subject must repeat them. Do 2 trials, even if 1st trial is successful.
Do a recall after 5 minutes.

	FACE	VELVET	CHURCH	DAISY	RED
1ST TRIAL					
2ND TRIAL					

NO POINTS

ATTENTION

Read list of digits (1 digit/ sec.). Subject has to repeat them in the forward order. [ ] 2 1 8 5 4
Subject has to repeat them in the backward order. [ ] 7 4 2

__/2

Read list of letters. The subject must tap with his hand at each letter A. No points if ≥ 2 errors
[ ] F B A C M N A A J K L B A F A K D E A A A J A M O F A A B

__/1

Serial 7 subtraction starting at 100. [ ] 93 [ ] 86 [ ] 79 [ ] 72 [ ] 65
4 or 5 correct subtractions: **3 pts**, 2 or 3 correct: **2 pts**, 1 correct: **1 pt**, 0 correct: **0**

__/3

LANGUAGE

Repeat: I only know that John is the one to help today. [ ]
The cat always hid under the couch when dogs were in the room. [ ]

__/2

Fluency: Name maximum number of words in one minute that begin with the letter F. [ ] _____ (N ≥ 11 words)

__/1

ABSTRACTION

Similarity between e.g. orange - banana = fruit [ ] train - bicycle [ ] watch - ruler

__/2

DELAYED RECALL

(MIS)		FACE	VELVET	CHURCH	DAISY	RED	
X3	Has to recall words WITH NO CUE	[ ]	[ ]	[ ]	[ ]	[ ]	Points for UNCUED recall only
X2	Category cue						MIS = ____/15
X1	Multiple choice cue						

Memory Index Score (MIS)

__/5

ORIENTATION

[ ] Date [ ] Month [ ] Year [ ] Day [ ] Place [ ] City

__/6

© MoCA Test Inc.
Created by Z. Nasreddine MD

www.mocacognition.com

Training and Certification are required to ensure accuracy.

MIS: /15
(Normal ≥ 26/30)
Add 1 point if ≤ 12 yr edu

TOTAL __/30

UCLA Version 3 Loneliness Scale

UCLA Version 3 Loneliness Scale

Instructions: The following statements describe how people sometimes feel. For each statement, please indicate how often you feel the way described by placing a check in the space provided. Here is an example: How often do you feel happy? If you never felt happy, you would check "never"; if you always feel happy, you would check "always."

	NEVER 1	RARELY 2	SOMETIMES 3	ALWAYS 4
*1. How often do you feel that you are "in tune" with the people around you?				
2. How often do you feel that you lack companionship?				
3. How often do you feel that there is no one you can turn to?				
4. How often do you feel alone?				
*5. How often do you feel part of a group of friends?				
*6. How often do you feel that you have a lot in common with the people around you?				
7. How often do you feel that you are no longer close to anyone?				
8. How often do you feel that your interests and ideas are not shared by those around you?				
*9. How often do you feel outgoing and friendly?				
*10. How often do you feel close to people?				
11. How often do you feel left out?				
12. How often do you feel that your relationships with others are not meaningful?				
13. How often do you feel that no one really knows you well?				
14. How often do you feel isolated from others?				
*15. How often do you feel you can find companionship when you want it?				
*16. How often do you feel that there are people who really understand you?				
17. How often do you feel shy?				
18. How often do you feel that people are around you but not with you?				
*19. How often do you feel that there are people you can talk to?				
*20. How often do you feel that there are people you can turn to?				

Scoring: Items that are asterisked should be reversed (i.e., 1 4, 2 3, 3 2, 4 1), and the scores for each item then summed together. Higher scores indicate greater degrees of loneliness. From Russell DW: UCLA Loneliness Scale (Version 3): reliability, validity, and factor structure, *J Pers Assess* 66:20-40, 1996.

IT-eHEALS

eHealth Literacy Scale

I would like to ask you for your opinion and about your experience using the Internet for health information. For each statement, tell me which response best reflects your opinion and experience *right now*.

1. How **useful** do you feel the Internet is in helping you in making decisions about your health?

o1	o2	o3	o4	o5
Not useful at all	Not useful	Unsure	Useful	Very Useful

2. How **important** is it for you to be able to access health resources on the Internet?

o1	o2	o3	o4	o5
Not important at all	Not important	Unsure	Important	Very important

3. I know **what** health resources are available on the Internet

1) o Strongly Disagree
2) o Disagree
3) o Undecided
4) o Agree
5) o Strongly Agree

4. I know **where** to find helpful health resources on the Internet

1) o Strongly Disagree
2) o Disagree
3) o Undecided
4) o Agree
5) o Strongly Agree

5. I know **how** to find helpful health resources on the Internet

1) o Strongly Disagree
2) o Disagree
3) o Undecided
4) o Agree
5) o Strongly Agree

6. I know **how to use** the Internet to answer my questions about health

1) o Strongly Disagree
2) o Disagree
3) o Undecided
4) o Agree
5) o Strongly Agree

7. I know how to use **the health information** I find on the Internet to help me

1) o Strongly Disagree
2) o Disagree
3) o Undecided
4) o Agree
5) o Strongly Agree

8. I have the skills I need to **evaluate** the health resources I find on the Internet

1) o Strongly Disagree
2) o Disagree
3) o Undecided
4) o Agree
5) o Strongly Agree

9. I can tell **high quality** health resources from **low quality** health resources on the Internet

1) o Strongly Disagree
2) o Disagree
3) o Undecided
4) o Agree
5) o Strongly Agree

10. I feel **confident** in using information from the Internet to make health decisions

1) o Strongly Disagree
2) o Disagree
3) o Undecided
4) o Agree
5) o Strongly Agree

Thank you!

** Note: Questions #1 and #2 are recommended as supplementary items for use with the eHEALS to understand consumer's interest in using eHealth in general. These items are not a formal part of theeHealth Literacy scale, which comprises questions #3-10.*

EQ-5D-5L

Under each heading, please tick the ONE box that best describes your health TODAY

MOBILITY

- I have no problems in walking about ☐
- I have slight problems in walking about ☐
- I have moderate problems in walking about ☐
- I have severe problems in walking about ☐
- I am unable to walk about ☐

SELF-CARE

- I have no problems washing or dressing myself ☐
- I have slight problems washing or dressing myself ☐
- I have moderate problems washing or dressing myself ☐
- I have severe problems washing or dressing myself ☐
- I am unable to wash or dress myself ☐

USUAL ACTIVITIES *(e.g. work, study, housework, family or leisure activities)*

- I have no problems doing my usual activities ☐
- I have slight problems doing my usual activities ☐
- I have moderate problems doing my usual activities ☐
- I have severe problems doing my usual activities ☐
- I am unable to do my usual activities ☐

PAIN / DISCOMFORT

- I have no pain or discomfort ☐
- I have slight pain or discomfort ☐
- I have moderate pain or discomfort ☐
- I have severe pain or discomfort ☐
- I have extreme pain or discomfort ☐

ANXIETY / DEPRESSION

- I am not anxious or depressed ☐
- I am slightly anxious or depressed ☐
- I am moderately anxious or depressed ☐
- I am severely anxious or depressed ☐
- I am extremely anxious or depressed ☐

VAS

To help people say how good or bad a health state is, we have drawn a scale (rather like a thermometer) on which the best state you can imagine is marked 100 and the worst state you can imagine is marked 0.

We would like you to indicate on this scale how good or bad your own health is today, in your opinion. Please do this by drawing a line from the box below to whichever point on the scale indicates how good or bad your health state is today.

Your own health state today

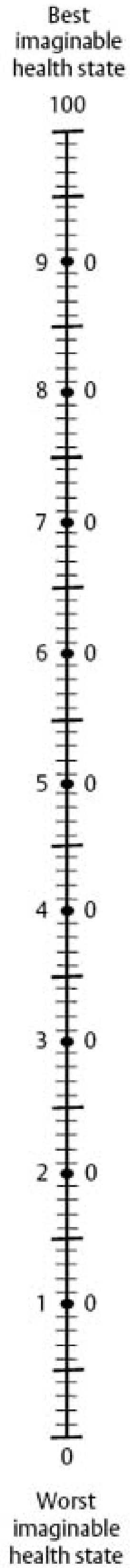

PWB - 18 items

Please indicate your degree of agreement to the following sentences.

1 = Strongly disagree   6 = Strongly agree

Maintaining close relationships has been difficult and frustrating for me.	1	2	3	4	5	6
The demands of everyday life often get me down.	1	2	3	4	5	6
In many ways, I feel disappointed about my achievements in life.	1	2	3	4	5	6
I like most aspects of my personality.	1	2	3	4	5	6
I have not experienced many warm and trusting relationships with others.	1	2	3	4	5	6
I judge myself by what I think is important, not by the values of what others think is important.	1	2	3	4	5	6
In general, I feel I am in charge of the situation in which I live.	1	2	3	4	5	6
I am quite good at managing the many responsibilities of my daily life.	1	2	3	4	5	6
I tend to be influenced by people with strong opinions.	1	2	3	4	5	6
I gave up trying to make big improvements or changes in my life a long time ago.	1	2	3	4	5	6
I think it is important to have new experiences that challenge how you think about yourself and the world.	1	2	3	4	5	6
I live life one day at a time and don't really think about the future.	1	2	3	4	5	6
I am not afraid to voice my opinions, even when they are in opposition to the opinions of most people.	1	2	3	4	5	6
Some people wander aimlessly through life, but I am not one of them.	1	2	3	4	5	6
Some people wander aimlessly through life, but I am not one of them.	1	2	3	4	5	6
For me, life has been a continuous process of learning, changing and growth.	1	2	3	4	5	6
People would describe me as a giving person, willing to share my time with others.	1	2	3	4	5	6
I sometimes feel as if I've done all there is to do in life.	1	2	3	4	5	6
When I look at the story of my life, I am pleased with how things have turned out.	1	2	3	4	5	6

System Usability Scale (SUS)

The following section relates to your feelings and thoughts that may rise while using the application/device. Please circle one in each of the following statements, the number that reflects your impressions. There is only one answer possible.

1 = Strongly disagree 5 = Strongly agree

I think that i would like to use the system more often.	1	2	3	4	5
I found the system unnecessarily complex.	1	2	3	4	5
I found the system was easily to handle.	1	2	3	4	5
I think I would need the help of a technical person to be able to use the system.	1	2	3	4	5
I found the different functions in the system were well integrated.	1	2	3	4	5
I think the system were too instable.	1	2	3	4	5
I can imagine the most of the people can easily learn to handle the system very quickly.	1	2	3	4	5
I found the system very uncomfortable to use.	1	2	3	4	5
I felt very safe while using the system.	1	2	3	4	5
I needed to learn a lot of things before I could get going with this system.	1	2	3	4	5

Unified Theory of Acceptance and Use of Technology (UTAUT)

Please circle one in each of the following statements, the number that reflects your impressions. There is only one answer possible.

1 = Strongly disagree   5 = Strongly agree

If I were to use the system, I would be afraid of making mistakes.	1	2	3	4	5	DK	Re
If I were to use the system, I would be afraid of breaking something.	1	2	3	4	5	DK	Re
The system scares me.	1	2	3	4	5	DK	Re
The system frightens me.	1	2	3	4	5	DK	Re
I think it is a good idea to use the system.	1	2	3	4	5	DK	Re
Using the system would make my life more interesting.	1	2	3	4	5	DK	Re
It is good to use the system.	1	2	3	4	5	DK	Re
I have everything I need to use the system correctly.	1	2	3	4	5	DK	Re
I know enough about the system to be able to use it correctly.	1	2	3	4	5	DK	Re
I think i will use the system in the next few days.	1	2	3	4	5	DK	Re
I am certain that I will use the system in the next few days.	1	2	3	4	5	DK	Re
I am planning to use the system in the next few days.	1	2	3	4	5	DK	Re
I believe the system can accommodate my needs.	1	2	3	4	5	DK	Re
I believe the system will only do what I need it to do at the moment.	1	2	3	4	5	DK	Re
I believe the system will help me when I feel it is necessary.	1	2	3	4	5	DK	Re
I enjoy that the system communicates with me.	1	2	3	4	5	DK	Re
I enjoy performing operations with the system.	1	2	3	4	5	DK	Re
I think the system is fun.	1	2	3	4	5	DK	Re

I think the system is interesting.	1	2	3	4	5	DK	Re
I think the system is boring.	1	2	3	4	5	DK	Re
I think I will learn to use the system quickly.	1	2	3	4	5	DK	Re
I think the system is easy to use.	1	2	3	4	5	DK	Re
I believe that I can use the system without help.	1	2	3	4	5	DK	Re
I think I could use the system with someone beside me.	1	2	3	4	5	DK	Re
I think I could use the system with a manual.	1	2	3	4	5	DK	Re
I think the system is useful for me.	1	2	3	4	5	DK	Re
It would be convenient for me to have the system.	1	2	3	4	5	DK	Re
I believe the system can help me in many activities.	1	2	3	4	5	DK	Re
I think the staff would be happy if I use the system.	1	2	3	4	5	DK	Re
I think I would make a good impression if I were to use the system.	1	2	3	4	5	DK	Re
I think I would believe the system if it gave me a warning.	1	2	3	4	5	DK	Re
I would follow the warnings the system would give me.	1	2	3	4	5	DK	Re

Follow-up (T2)

Identification code:
Date of compilation:
Name of the interviewer:

Montreal Cognitive Assessment (MoCA)

MoCA COGNITIVE ASSESSMENT — Version 8.1 English

Name:
Education: Date of birth:
Sex: DATE:

	POINTS
**VISUOSPATIAL / EXECUTIVE**	

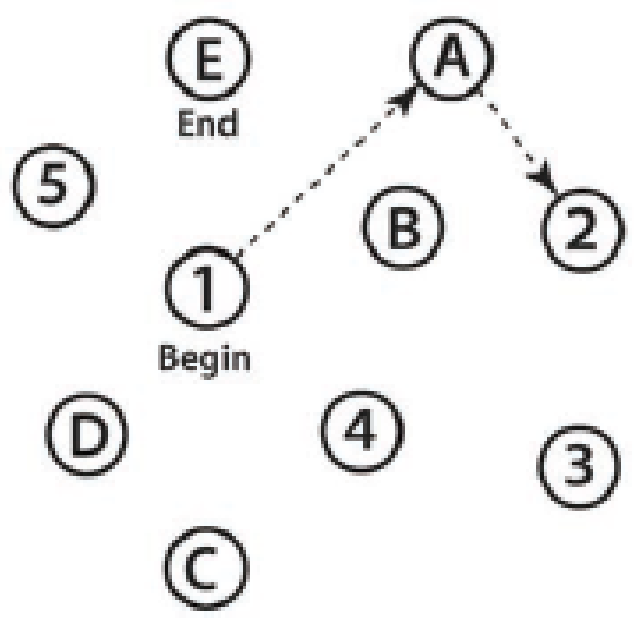

[ ]

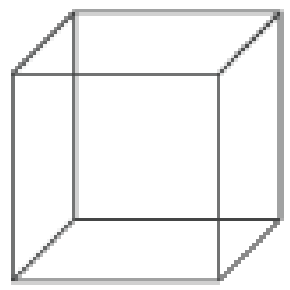

Copy cube

[ ]

Draw CLOCK (Ten past eleven)
(3 points)

[ ] Contour [ ] Numbers [ ] Hands

___/5

NAMING

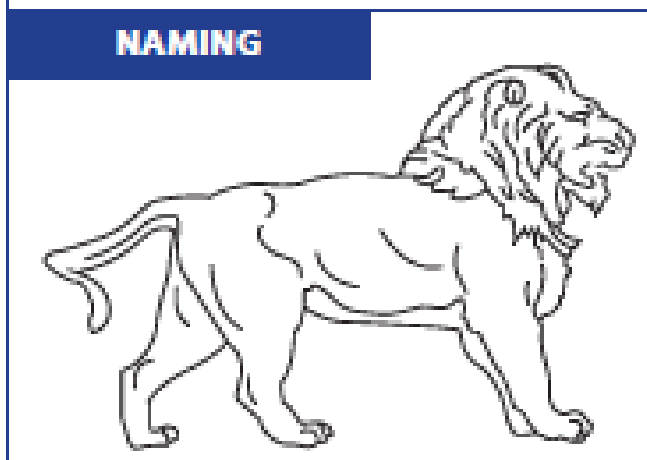

[ ]

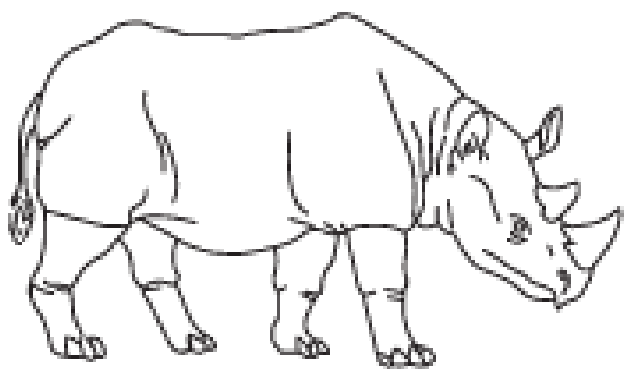

[ ]

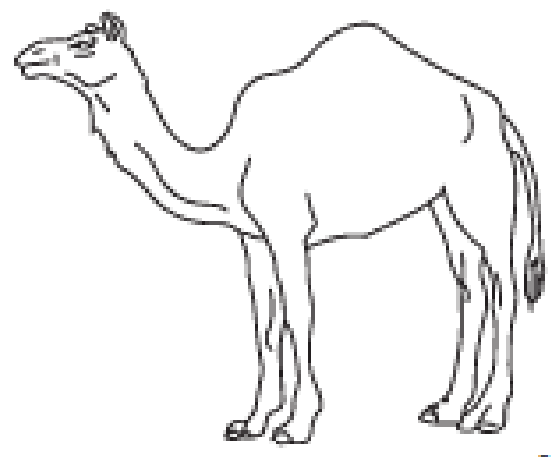

[ ]

___/3

MEMORY

Read list of words, subject must repeat them. Do 2 trials, even if 1st trial is successful. Do a recall after 5 minutes.

	FACE	VELVET	CHURCH	DAISY	RED
1ST TRIAL					
2ND TRIAL					

NO POINTS

ATTENTION

Read list of digits (1 digit/ sec.). Subject has to repeat them in the forward order. [ ] 2 1 8 5 4
Subject has to repeat them in the backward order. [ ] 7 4 2

___/2

Read list of letters. The subject must tap with his hand at each letter A. No points if ≥ 2 errors
[ ] F B A C M N A A J K L B A F A K D E A A A J A M O F A A B

___/1

Serial 7 subtraction starting at 100. [ ] 93 [ ] 86 [ ] 79 [ ] 72 [ ] 65
4 or 5 correct subtractions: **3 pts**, 2 or 3 correct: **2 pts**, 1 correct: **1 pt**, 0 correct: **0**

___/3

LANGUAGE

Repeat: I only know that John is the one to help today. [ ]
The cat always hid under the couch when dogs were in the room. [ ]

___/2

Fluency: Name maximum number of words in one minute that begin with the letter F. [ ] _____ (N ≥ 11 words)

___/1

ABSTRACTION

Similarity between e.g. orange - banana = fruit [ ] train - bicycle [ ] watch - ruler

___/2

DELAYED RECALL

(MIS)		FACE	VELVET	CHURCH	DAISY	RED	
	Has to recall words WITH NO CUE	[ ]	[ ]	[ ]	[ ]	[ ]	Points for UNCUED recall only
X3							
X2	Category cue						MIS = ____/15
X1	Multiple choice cue						

Memory Index Score (MIS)

___/5

ORIENTATION

[ ] Date [ ] Month [ ] Year [ ] Day [ ] Place [ ] City

___/6

© MoCA Test Inc.
Created by Z. Nasreddine MD

www.mocacognition.com

Training and Certification are required to ensure accuracy.

MIS: /15
(Normal ≥ 26/30)
Add 1 point if ≤ 12 yr edu

TOTAL ___/30

UCLA Version 3 Loneliness Scale

UCLA Version 3 Loneliness Scale

Instructions: The following statements describe how people sometimes feel. For each statement, please indicate how often you feel the way described by placing a check in the space provided. Here is an example: How often do you feel happy? If you never felt happy, you would check "never"; if you always feel happy, you would check "always."

	NEVER<br>1	RARELY<br>2	SOMETIMES<br>3	ALWAYS<br>4
*1. How often do you feel that you are "in tune" with the people around you?				
2. How often do you feel that you lack companionship?				
3. How often do you feel that there is no one you can turn to?				
4. How often do you feel alone?				
*5. How often do you feel part of a group of friends?				
*6. How often do you feel that you have a lot in common with the people around you?				
7. How often do you feel that you are no longer close to anyone?				
8. How often do you feel that your interests and ideas are not shared by those around you?				
*9. How often do you feel outgoing and friendly?				
*10. How often do you feel close to people?				
11. How often do you feel left out?				
12. How often do you feel that your relationships with others are not meaningful?				
13. How often do you feel that no one really knows you well?				
14. How often do you feel isolated from others?				
*15. How often do you feel you can find companionship when you want it?				
*16. How often do you feel that there are people who really understand you?				
17. How often do you feel shy?				
18. How often do you feel that people are around you but not with you?				
*19. How often do you feel that there are people you can talk to?				
*20. How often do you feel that there are people you can turn to?				

Scoring: Items that are asterisked should be reversed (i.e., 1 4, 2 3, 3 2, 4 1), and the scores for each item then summed together. Higher scores indicate greater degrees of loneliness. From Russell DW: UCLA Loneliness Scale (Version 3): reliability, validity, and factor structure, *J Pers Assess* 66:20-40, 1996.

eHealth Literacy Scale

I would like to ask you for your opinion and about your experience using the Internet for health information. For each statement, tell me which response best reflects your opinion and experience *right now*.

1. How **useful** do you feel the Internet is in helping you in making decisions about your health?

o1	o2	o3	o4	o5
Not useful at all	Not useful	Unsure	Useful	Very Useful

2. How **important** is it for you to be able to access health resources on the Internet?

o1	o2	o3	o4	o5
Not important at all	Not important	Unsure	Important	Very important

3. I know **what** health resources are available on the Internet

1) o Strongly Disagree
2) o Disagree
3) o Undecided
4) o Agree
5) o Strongly Agree

4. I know **where** to find helpful health resources on the Internet

1) o Strongly Disagree
2) o Disagree
3) o Undecided
4) o Agree
5) o Strongly Agree

5. I know **how** to find helpful health resources on the Internet

1) o Strongly Disagree
2) o Disagree
3) o Undecided
4) o Agree
5) o Strongly Agree

6. I know **how to use** the Internet to answer my questions about health

1) o Strongly Disagree
2) o Disagree
3) o Undecided
4) o Agree
5) o Strongly Agree

7. I know how to use **the health information** I find on the Internet to help me

1) o Strongly Disagree
2) o Disagree
3) o Undecided
4) o Agree
5) o Strongly Agree

8. I have the skills I need to **evaluate** the health resources I find on the Internet

1) o Strongly Disagree
2) o Disagree
3) o Undecided
4) o Agree
5) o Strongly Agree

9. I can tell **high quality** health resources from **low quality** health resources on the Internet

1) o Strongly Disagree
2) o Disagree
3) o Undecided
4) o Agree
5) o Strongly Agree

10. I feel **confident** in using information from the Internet to make health decisions

1) o Strongly Disagree
2) o Disagree
3) o Undecided
4) o Agree
5) o Strongly Agree

Thank you!

** Note: Questions #1 and #2 are recommended as supplementary items for use with the eHEALS to understand consumer's interest in using eHealth in general. These items are not a formal part of theeHealth Literacy scale, which comprises questions #3-10.*

EQ-5D-5L

Under each heading, please tick the ONE box that best describes your health TODAY

MOBILITY

I have no problems in walking about	☐
I have slight problems in walking about	☐
I have moderate problems in walking about	☐
I have severe problems in walking about	☐
I am unable to walk about	☐

SELF-CARE

I have no problems washing or dressing myself	☐
I have slight problems washing or dressing myself	☐
I have moderate problems washing or dressing myself	☐
I have severe problems washing or dressing myself	☐
I am unable to wash or dress myself	☐

USUAL ACTIVITIES *(e.g. work, study, housework, family or leisure activities)*

I have no problems doing my usual activities	☐
I have slight problems doing my usual activities	☐
I have moderate problems doing my usual activities	☐
I have severe problems doing my usual activities	☐
I am unable to do my usual activities	☐

PAIN / DISCOMFORT

I have no pain or discomfort	☐
I have slight pain or discomfort	☐
I have moderate pain or discomfort	☐
I have severe pain or discomfort	☐
I have extreme pain or discomfort	☐

ANXIETY / DEPRESSION

I am not anxious or depressed	☐
I am slightly anxious or depressed	☐
I am moderately anxious or depressed	☐
I am severely anxious or depressed	☐
I am extremely anxious or depressed	☐

VAS

To help people say how good or bad a health state is, we have drawn a scale (rather like a thermometer) on which the best state you can imagine is marked 100 and the worst state you can imagine is marked 0.

We would like you to indicate on this scale how good or bad your own health is today, in your opinion. Please do this by drawing a line from the box below to whichever point on the scale indicates how good or bad your health state is today.

Your own health state today

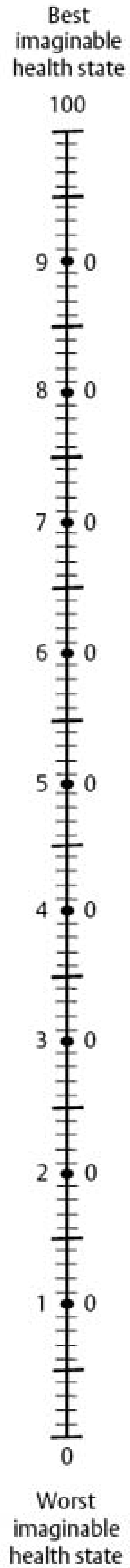

PWB - 18 items

Please indicate your degree of agreement to the following sentences.

1 = Strongly disagree 6 = Strongly agree

Maintaining close relationships has been difficult and frustrating for me.	1	2	3	4	5	6
The demands of everyday life often get me down.	1	2	3	4	5	6
In many ways, I feel disappointed about my achievements in life.	1	2	3	4	5	6
I like most aspects of my personality.	1	2	3	4	5	6
I have not experienced many warm and trusting relationships with others.	1	2	3	4	5	6
I judge myself by what I think is important, not by the values of what others think is important.	1	2	3	4	5	6
In general, I feel I am in charge of the situation in which I live.	1	2	3	4	5	6
I am quite good at managing the many responsibilities of my daily life.	1	2	3	4	5	6
I tend to be influenced by people with strong opinions.	1	2	3	4	5	6
I gave up trying to make big improvements or changes in my life a long time ago.	1	2	3	4	5	6
I think it is important to have new experiences that challenge how you think about yourself and the world.	1	2	3	4	5	6
I live life one day at a time and don't really think about the future.	1	2	3	4	5	6
I am not afraid to voice my opinions, even when they are in opposition to the opinions of most people.	1	2	3	4	5	6
Some people wander aimlessly through life, but I am not one of them.	1	2	3	4	5	6
Some people wander aimlessly through life, but I am not one of them.	1	2	3	4	5	6
For me, life has been a continuous process of learning, changing and growth.	1	2	3	4	5	6
People would describe me as a giving person, willing to share my time with others.	1	2	3	4	5	6
I sometimes feel as if I've done all there is to do in life.	1	2	3	4	5	6
When I look at the story of my life, I am pleased with how things have turned out.	1	2	3	4	5	6

System Usability Scale (SUS)

The following section relates to your feelings and thoughts that may rise while using the application/device. Please circle one in each of the following statements, the number that reflects your impressions. There is only one answer possible.

1 = Strongly disagree 5 = Strongly agree

I think that i would like to use the system more often.	1	2	3	4	5
I found the system unnecessarily complex.	1	2	3	4	5
I found the system was easily to handle.	1	2	3	4	5
I think I would need the help of a technical person to be able to use the system.	1	2	3	4	5
I found the different functions in the system were well integrated.	1	2	3	4	5
I think the system were too instable.	1	2	3	4	5
I can imagine the most of the people can easily learn to handle the system very quickly.	1	2	3	4	5
I found the system very uncomfortable to use.	1	2	3	4	5
I felt very safe while using the system.	1	2	3	4	5
I needed to learn a lot of things before I could get going with this system.	1	2	3	4	5

Unified Theory of Acceptance and Use of Technology (UTAUT)

Please circle one in each of the following statements, the number that reflects your impressions. There is only one answer possible.

1 = Strongly disagree 5 = Strongly agree

If I were to use the system, I would be afraid of making mistakes.	1	2	3	4	5	DK	Re
If I were to use the system, I would be afraid of breaking something.	1	2	3	4	5	DK	Re
The system scares me.	1	2	3	4	5	DK	Re
The system frightens me.	1	2	3	4	5	DK	Re
I think it is a good idea to use the system.	1	2	3	4	5	DK	Re
Using the system would make my life more interesting.	1	2	3	4	5	DK	Re
It is good to use the system.	1	2	3	4	5	DK	Re
I have everything I need to use the system correctly.	1	2	3	4	5	DK	Re
I know enough about the system to be able to use it correctly.	1	2	3	4	5	DK	Re
I think i will use the system in the next few days.	1	2	3	4	5	DK	Re
I am certain that I will use the system in the next few days.	1	2	3	4	5	DK	Re
I am planning to use the system in the next few days.	1	2	3	4	5	DK	Re
I believe the system can accommodate my needs.	1	2	3	4	5	DK	Re
I believe the system will only do what I need it to do at the moment.	1	2	3	4	5	DK	Re
I believe the system will help me when I feel it is necessary.	1	2	3	4	5	DK	Re
I enjoy that the system communicates with me.	1	2	3	4	5	DK	Re
I enjoy performing operations with the system.	1	2	3	4	5	DK	Re
I think the system is fun.	1	2	3	4	5	DK	Re

I think the system is interesting.	1	2	3	4	5	DK	Re
I think the system is boring.	1	2	3	4	5	DK	Re
I think I will learn to use the system quickly.	1	2	3	4	5	DK	Re
I think the system is easy to use.	1	2	3	4	5	DK	Re
I believe that I can use the system without help.	1	2	3	4	5	DK	Re
I think I could use the system with someone beside me.	1	2	3	4	5	DK	Re
I think I could use the system with a manual.	1	2	3	4	5	DK	Re
I think the system is useful for me.	1	2	3	4	5	DK	Re
It would be convenient for me to have the system.	1	2	3	4	5	DK	Re
I believe the system can help me in many activities.	1	2	3	4	5	DK	Re
I think the staff would be happy if I use the system.	1	2	3	4	5	DK	Re
I think I would make a good impression if I were to use the system.	1	2	3	4	5	DK	Re
I think I would believe the system if it gave me a warning.	1	2	3	4	5	DK	Re
I would follow the warnings the system would give me.	1	2	3	4	5	DK	Re
